# Neural underpinnings of the interplay between actual touch and action imagination in social contexts

**DOI:** 10.3389/fnhum.2023.1274299

**Published:** 2024-01-11

**Authors:** Yumna Ali, Veronica Montani, Paola Cesari

**Affiliations:** Department of Neuroscience, Biomedicine and Movement Sciences, University of Verona, Verona, Italy

**Keywords:** action imagination, force production, social touch, motor cortex, transcranial magnetic stimulation (TMS)

## Abstract

While there is established evidence supporting the involvement of the sense of touch in various actions, the neural underpinnings of touch and action interplay in a social context remain poorly understood. To prospectively investigate this phenomenon and offer further insights, we employed a combination of motor and sensory components by asking participants to imagine exerting force with the index finger while experiencing their own touch, the touch of one another individual, the touch of a surface, and no touch. Based on the assumption that the patterns of activation in the motor system are similar when action is imagined or actually performed, we proceeded to apply a single-pulse transcranial magnetic stimulation over the primary motor cortex (M1) while participants engaged in the act of imagination. Touch experience was associated with higher M1 excitability in the presence and in the absence of force production imagination, but only during force production imagination M1 excitability differed among the types of touch: both biological sources, the self-touch and the touch of one other individual, elicited a significant increase in motor system activity when compared to touching a non-living surface or in the absence of touch. A strong correlation between individual touch avoidance questionnaire values and facilitation in the motor system was present while touching another person, indicating a social aspect for touch in action. The present study unveils the motor system correlates when the sensory/motor components of touch are considered in social contexts.

## Introduction

The influence of physical touch permeates our entire life by deeply influencing how we act. Through touch, we acquire information about the surface’s physical components like hardness, roughness, softness, and temperature. By engaging in tactile experiences, we regulate the amount of pressure exerted while grasping objects. Self-touch and touching other individuals allow the processing of information about the self and the body (Martin, 1993). The experience of touch encompasses a complex interplay of cognitive and emotional factors, and pre-existing knowledge and expectations shape the individual’s perception leading to the emergence of personal traits (Bensmaia and Hollins, 2003; Haans and IJsselsteijn, 2006; Jones, 2016).

Touch can be passively sensed or actively perceived through action. While passive touch relies on the activation of cutaneous receptors, active touch receives additional input from the kinesthetic and proprioceptive senses, resulting in a different pattern of brain activation (e.g., Simões-Franklin et al., 2011). Since the seminal work of Gibson (Gibson, 1962; West and Gibson, 1969) much effort has been put into distinguishing the effects that those different exploratory procedures exert on object perception (Sonneveld and Schifferstein, 2008; Smith and Collins, 2009; Simões-Franklin et al., 2011; Ackerley et al., 2012; Fernandes and Albuquerque, 2012). The basic ability to discriminate different textures appears substantially equivalent in passive and active touch (Lederman, 1981; Heller, 1989; Verrillo et al., 1999). However, during active touch, specialized exploratory movements, and adjustment of movement parameters such as force, displacement, and related derivatives, allow to optimize perceptual precision (Lederman and Klatzky, 1987; Chapman, 1994; Srinivasan and LaMotte, 1995; Louw et al., 2000; Gamzu and Ahissar, 2001; Giachritsis et al., 2009; Kaim and Drewing, 2011; Drewing, 2012; Lezkan and Drewing, 2014; Metzger et al., 2018). In addition, more and more attention is devoted to the fact that to generate the appropriate percept, the afferent signal provided by the sensory receptors is integrated with the predictions of the sensory consequences of one’s own actions (generated as a consequence of the motor command, i.e., internal forward model or efference copy). In this way, not only the brain may extract the tactile signal more efficiently (Willemet et al., 2021), but the information about the perceptual context that is provided by the internal model can be critical to solving more complex tasks such as ambiguous shapes discrimination (Verrillo et al., 1999; Smith et al., 2009).

The tight functional coupling between touch and action is supported by strong neural interconnections between the somatic and the motor systems. After the initial processing within the primary somatosensory cortex (S1), tactile information travels through the secondary somatosensory cortex (S2), the posterior parietal cortex (PPC), and the primary motor (M1) cortex, (see review Delhaye et al., 2016). The primary motor cortex (M1) and primary somatosensory cortex (S1) are directly interconnected (Haegens et al., 2011; Ifft et al., 2013; Schwarz et al., 2014; Umeda et al., 2019). M1 and S1 work together to integrate tactile and proprioceptive feedback for skilled movements of the hand (Salimi et al., 1999; Gardner et al., 2007). Indeed, when the action of grasping is performed, a significant proportion of S1 neurons discharge in response (Sinclair and Burton, 1991), and some S1 neurons are sensitive to force direction (Fortier-Poisson and Smith, 2015; Fortier-Poisson et al., 2015). On the other hand, surface texture can be encoded by neurons in M1 (Jiang et al., 2018).

An experimental approach aimed to differentiate the neural mechanisms underlying perception from those involved in the action is applying motor imagery. Participants imagine performing an action while receiving sensory input, without physically executing any movement. The prevailing theoretical framework assumes that imagined actions are an internal simulation of actual movements (Jeannerod, 1994; Jeannerod and Decety, 1995) and that the cognitive mechanisms involved in action generation, perception, and imagination share a common network (Prinz, 1997; Hommel, 2009; Cesari et al., 2011). Even though the mechanisms behind imagined movements are still not fully comprehended, empirical findings indicate that these mental representations exhibit physiological and temporal patterns that resemble those of actual movements (Bufalari et al., 2010). Moreover, pathophysiological constraints seem to similarly affect both imagined and executed movements (Sirigu et al., 1996; Ridderinkhof and Brass, 2015).

Based on this method, few studies have examined the role of tactile information in controlling action (Mizuguchi et al., 2009, 2011; Ali et al., 2023), The corticospinal excitability of M1 during action imagination is enhanced by the real touch of the object involved in the action compared to the condition in which no touch is present (Mizuguchi et al., 2009, 2011), suggesting a facilitatory effect of touch on the motor system. Recently, we demonstrated that this excitability is muscle-specific (Ali et al., 2023). In our previous study, participants imagined producing different amounts of force with the index finger while touching or not a rigid surface. The facilitatory effect of touch scaled properly with the amount of force “exerted,” but crucially, the effect was restricted to the body part that would be involved in the execution of the action (Ali et al., 2023).

The ability to discriminate physical properties through touch is accompanied by the integration and processing of affective and socially relevant information. Touch received from other individuals, i.e., social touch, is crucial for the development of the individual’s social cognition, and then has a profound impact throughout the entire lifespan (Jönsson, 2017; Cascio et al., 2019). Affective touch is mediated by a specialized sub-modality of touch, the c-touch system (McGlone et al., 2014), and tactile stimulation from another person is associated with a distinct pattern of cortical activation compared to self-touch (Boehme et al., 2019, Boehme and Olausson, 2022). For example, (Boehme et al., 2019) used fMRI, behavioral testing, and somatosensory-evoked potentials (SEPs) measured at the spinal and cortical levels, to examine the difference between the sensation of touching oneself and the sensation of being touched by another person. Stimulation from another person activated several areas including the somatosensory cortex, insula, superior temporal gyrus, supramarginal gyrus, striatum, amygdala, cerebellum, and prefrontal cortex. In contrast, self-touch was associated with deactivation in the insula, anterior cingulate cortex, superior temporal gyrus, amygdala, parahippocampal gyrus, prefrontal areas, and brain areas encoding low-level sensory representations. Somatosensory Evoked Potentials (SEP) analysis showed reduced cortical amplitudes during self-touch, and in contrast, shorter latencies for other touch. Crucially, during self-touch, the functional connectivity between the sensorimotor cortex and the insula was accompanied by an elevated threshold for detecting additional tactile stimuli. The discrimination of self- or other-touch would be based on a mechanism of attenuation in which the internal forward model or efference copy allows the brain to predict the sensory consequences of one’s own action (see e.g., Boehme et al., 2019; Boehme and Olausson, 2022). Subsequently, the brain can reduce or not these sensations depending on the task at hand (Juravle et al., 2017). However, the neural processes that mediate the attenuation or non-attenuation remain to be understood.

How exactly affective touch can influence action control is relatively unexplored. The amplitude of the readiness potential was modulated by the pleasantness of the stimuli to be grasped (compatible vs. incompatible, de Oliveira et al., 2012), or by the emotional context induced before performing a pleasant action (gently caressing a soft cloth, Campagnoli et al., 2015). The facilitatory effect on the motor cortex induced by the pleasant emotional context before the action, suggests that anticipating the outcomes of an action involves assessing the emotional value of a stimulus one is going to engage with and this is in line with the hypothesis of a general facilitatory effect of touch (Mizuguchi et al., 2009, 2011; Ali et al., 2023). However, because of the relevance of social interaction, and the role of affective touch in social contexts (Dunbar, 2010), it is plausible that the influence of affective touch on action control might be based on a different mechanism.

Based on this framework, the aim of the present study was to clarify the neural mechanisms of the difference between the self and other people’s touch while the motor system is activated. Above we have briefly reviewed the thigh coupling between touch and action, and in particular how sensorimotor control and haptic exploration depend on the ability of the touch system to discriminate physical attributes. Much less is known about how affective touch can influence action control. We investigated whether the motor system resonates selectively for a task parameter such as force production imagination when different conditions of tactile stimuli are given (Cesari et al., 2011; Kilteni et al., 2018; Fukumoto et al., 2021). Specifically, we examined whether the activity of the motor system is differently modulated when an individual imagines producing and not producing force with her/his index finger while having the hand touching different surfaces: Self-Touch (put in contact his/her right with the left hand’s index fingertips), We-Touch (when subject receive the touch on his index finger from the index finger’s tip of one other person), Surface-Touch (touch a hard surface with his/her index fingertip), and No-Touch (do not have the index fingertip or other parts of the hand in touch with anything). To measure motor cortex activity, a single pulse Transcranial Magnetic Stimulation (TMS) was delivered while participants imagined producing ∼3N and not producing force (<∼1N) with the index finger of their right hand. As the target muscle, we selected the First Dorsal Interosseous (FDI) which is involved in the flexion-abduction of the index finger, while as the control muscle, the Abductor Digiti Minimi (ADM) since it is involved in the flexion-abduction of the little finger.

Considering the touch condition, we hypothesized that the motor system will show higher cortico-spinal activation when biological sources are touched, i.e., when individuals are either touching themselves and when touching someone else, as compared to when they are touching non-living sources, or not touching at all. However, when social interaction is included, we might expect to find higher cortico-spinal activation when touching someone else compared to the self-touch condition since it has been shown that the processing of social information undergoes distinctive and privileged processing mechanisms (de Oliveira et al., 2012; Souza et al., 2012; Campagnoli et al., 2015; Boehme and Olausson, 2022; Grichtchouk et al., 2022). Alternatively, participants may perceive self-generated tactile stimulation as relatively weaker, when compared to external tactile stimulation (Kilteni et al., 2018). In this case, participants could enhance the level of applied force, resulting in higher Motor Evoked Potentials (MEPs) in the Self-Touch condition. When considering the MEPs modulation based on the level of force imagined, we expected to replicate the results obtained in previous works (Helm et al., 2015; Ali et al., 2023), i.e., higher activation when individuals imagine producing higher force as compared to lower force. Here we asked individuals to imagine producing on the provided surface ∼3N of force (from now on referred as ∼3N condition), and to imagine the digit lying on the provided surface without imagining force production (from now on referred as <∼1N condition). For the interaction between the touch and the force conditions, we were expecting to find a generalized amplified effect of touch during the imagination of force production.

## Materials and methods

### Participants

The study included twenty healthy volunteers, 10 men, and 10 women, ranging in age from 18 to 26 years (mean age = 21.5 years, SD = 1.71 years). None of the participants were aware of the study’s purpose, and none of them had any neurological, psychiatric, or other health conditions. They also didn’t experience any negative effects from TMS (Rossi et al., 2021). There were no complaints of pain or adverse effects during TMS, and none were observed. Before entering the lab, each participant signed an informed consent form. The local ethics commission authorized the procedures, which adhered to the ethical standards set forth in the Declaration of Helsinki from 1964.

### Equipment

A biphasic single TMS pulse was applied using a figure-of-eight coil; the outside diameter of each wing was 110 mm (STM 9000 magnetic stimulator, Ates-EBNeuro, Italy). The coil was positioned on an extended arm at a 45° angle to the sagittal axis on the left side of the head, tangential to the skull (Brasil-Neto et al., 1992). The coil was moved laterally in small steps to the vertex in the left hemisphere and TMS pulses were given once stable motor evoked potential (MEP) amplitudes were evoked in the relaxed FDI (Jong et al., 2009). The resting motor threshold is the lowest stimulus intensity that can elicit MEPs in the muscles with an amplitude of at least 50 V in at least five out of ten trials (rMT). This measurement was used to identify the best area of the scalp for FDI. Throughout the experiment, the amount of stimulation was set at 120% rMT (Hallett, 2000).

### Procedure

The task required to imagine producing or not forces while lying the right hand’s index finger on the various surfaces defining the different touch conditions. The subjects were asked to maintain their right arm relaxed on their lap, which was supported by a hard surface while sitting in a chair. Because the compatibility of the hand position could impact the MEP amplitude (Vargas et al., 2004; Casetta et al., 2020), the hand remained in the same position throughout the entire experiment, to ensure a posture compatible with the imaged action. The experiment was recorded in a block based on conditions where each block of four touch conditions contains two levels of force counterbalanced among subjects. The task was to imagine with closed eyes on two levels i.e., <∼1 and ∼3N of forces. Fifteen trials of TMS stimulation were delivered for each combination of force condition and the types of touch condition i.e., Self-Touch when the subject receives the touch of one other person (We-Touch), Surface Touch, and without any touch (No-Touch). A total of 120 trials were recorded from each participant.

**FIGURE 1:**
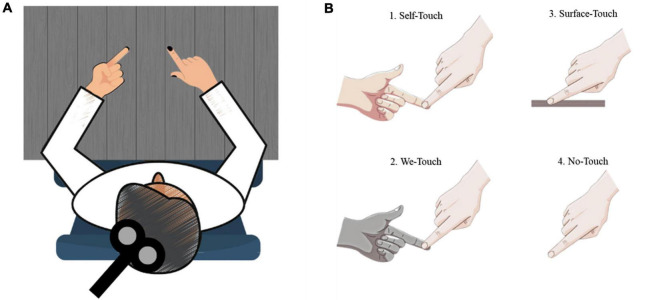
Schematic illustration of test situations. **(A)** Overview; **(B)** 1. Self-touch - Individual touching own index finger, 2. We touch - Another person touching the participant’s index finger, 3. Surface touch - Surface provided beneath participant’s index finger, 4. No touch - No touch provided.

A training session was conducted prior to the start of the imagination task. Participants were instructed to practice, utilizing the tip of their index fingers, to produce a specific amount of force on the balance scale. During the training session, the experimenter provided guidance to the subjects regarding the timing of the task, including when to initiate the action, the duration of force application, and when to release the force. Once the participants became accustomed to the timing of the task, a recorded voice signaled the start and end of each trial. The participants were instructed to actively remember their experiences of producing force during the training session, with the goal of being able to mentally simulate the same experiences during the experimental trials. Following the training session, participants were instructed to maintain a seated position with their arms at rest and their heads facing forward for the entire duration of the data collection (see Figure 1).

As previously mentioned, the experimental task required participants to engage in mental imagery of force production (3N) and no force production (>∼1N) using their index fingers. The magnitude of the imagined force was matched to the force levels practiced during the training session. Throughout the entire experiment, participants were not provided with any feedback regarding their performance. Fatigue was carefully monitored, and rest periods were incorporated every 30 trials to mitigate any potential fatigue effects. Each trial involved the delivery of a single transcranial magnetic stimulation (TMS) pulse. To avoid any potential effect associated with expectation, TMS was applied at random delays (selected among intervals of 300, 450, 600, 750, or 1000 ms, balanced across trials) after the vocal instruction. The inter-trial interval, approximately 5−10 s, was designed to prevent the accumulation of brain activity from one TMS pulse to the next, following the guidelines proposed by Wassermann et al. (2008). The entire experimental session for each participant lasted approximately 80 min, encompassing multiple trials and TMS pulses. At the end of the experiment, participants were asked to fill out some questionnaires, the Vividness of Visual Imagery Questionnaire (VVIQ) to test the effectiveness of their imagery (Marks, 1973), and the Touch Avoidance measure, and touch avoidance questionnaire (Casetta et al., 2020) to assess the relevant personality dimensions.

### Statistical analysis

We employed mixed-effect multiple regression modeling (Gelman and Hill, 2006; Yu et al., 2022) to examine the impact of touch, force, and muscle on motor-evoked potential (MEP) amplitude. The model incorporated three fixed effects (touch, force, and muscle) and their interaction. Touch had four levels (you-touch, me-touch, surface-touch, and no-touch), the force had two levels (<∼1 Newton, ∼3 Newton), and muscle had two levels (FDI, ADM). The random structure of the model included by-subject random intercepts and by-subject random slopes for the force factor. To account for multiple comparisons, we applied the Bonferroni procedure for *p*-value correction (Simes, 1986). Observations with standardized residuals exceeding 2.5 standard deviations from zero were excluded (3.90% of the data), following guidelines by Krajewski and Matthews (2010).

In addition, we assessed whether there was an association between the individual differences between the Self-Touch and We-Touch conditions (that we refer to as the “Self-We Touch effect”), and the touch avoidance measure obtained from the two questionnaires. As an operational measure of the “Self-We Touch effect,” we computed the ratio between Self-Touch and We-Touch, in the ∼3N force condition for FDI and ADM separately. Statistical analysis was conducted using the lme4 package (Bates et al., 2015), the afex package (Singmann et al., 2022), and the emmeans package (Lenth, 2022) in the R environment (R Core Team, 2022)

## Results

Based on the participants’ overall VVIQ ratings, which were a 2.16 (SD = 0.7) average score for participants with open eyes and 2.26 (SD = 0.67) for closed eyes condition, all participants were referred to the group of “good visualizers” (Marks, 1973). All participants successfully performed force production imagination (3N) and no force production imagination (<∼1N) for the 4 levels of touch (Self-Touch, We-Touch, Surface-Touch, and No-Touch) by using two muscles i.e., FDI and ADM. None of the participants reported discomfort during the stimulations.

**FIGURE 2:**
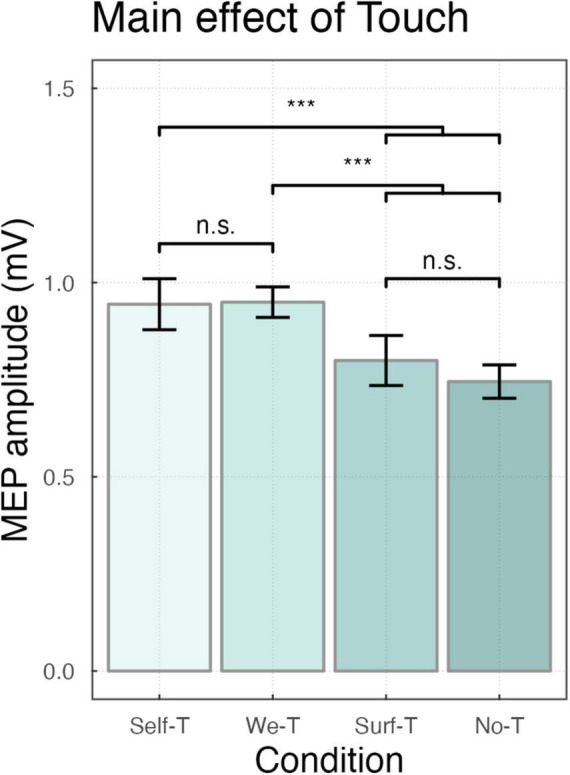
Bar plot showing the main effect of Touch ***P < 0.001, with n.s. indicating non-significance. Error bars represent the standard error of the means adjusted to correctly reflect the variance in the within-subject design (Morey, 2008).

Considering the motor-evoked potentials, the main effect of Force was significant (F_ (1_,_18)_ = 18.97, *p* < 0.001) indicating that, overall, MEPs were smaller for <∼1N (*M* = 0.78, SD = 0.82) compared to ∼3N (*M* = 1.02, SD = 1.01). The main effect of Condition was significant (F_ (3_,_4485)_ = 26.58, *p* < 0.001), *Post-hoc* comparisons using Bonferroni correction indicated that, overall, MEPs were larger for both Self-Touch (*M* = 1.00, SD = 0.83, *p* < 0.001), and We-Touch (*M* = 1.00, SD = 0.68, *p* < 0.001) compared to Surface-Touch (*M* = 0.83, SD = 0.64), and, for both Self-Touch (*p* < 0.001) and We-Touch (*p* < 0.001) compared to No-Touch (*M* = 0.77, SD = 0.61). MEPs for Self-Touch did not significantly differ from MEPs for We-Touch (*p* = 1.00), and MEPs for Surface-Touch did not significantly differ from MEPs for No-Touch (*p* = 0.72). The main effect of Muscle (F_ (1_,_4488)_ = 840.69, *p* < 0.001) was significant, indicating that, overall, MEPs were larger for FDI (*M* = 1.22, SD = 0.80) compared to ADM (*M* = 0.57, SD = 0.60) (see Figure 2).

**FIGURE 3:**
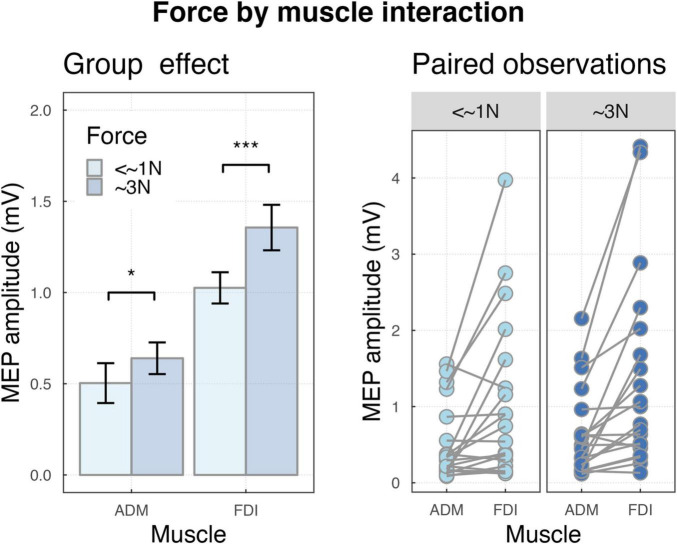
**(Left)** Bar plot showing the group effects of force by muscle interaction ***P < 0.001 and *P < 0.05. Error bars represent the standard error of the means adjusted to correctly reflect the variance in the within-subject design (Morey, 2008). **(Right)** Paired observation of two muscles ADM and FDI in forces.

The two-way interaction force by muscles was significant (F_(1,4490)_ = 19.18, *p* < 0.001), indicating that, MEPs were smaller for <∼1N compared to ∼3N for FDI (*M* = 1.03, SD = 0.38, *M* = 1.36, SD = 0.56, *p* < 0.001) and ADM (*M* = 0.5, SD = 0.49, *M* = 0.64, SD = 0.39, *p* = 0.03), but the difference was larger for FDI (see Figure 3).

**FIGURE 4:**
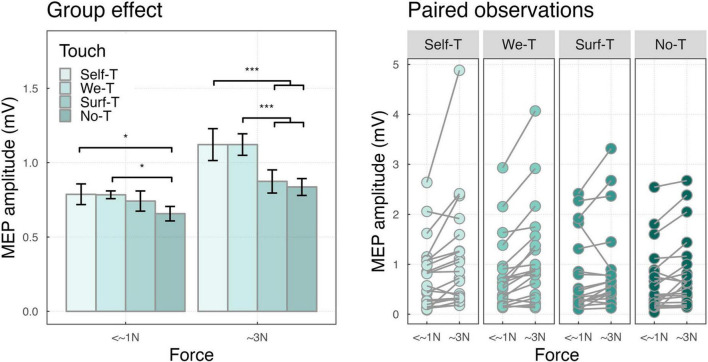
**(Left)** Bar plot showing the group effects of touch-by-force interaction ***P < 0.001 and *P < 0.05. Error bars represent the standard error of the means adjusted to correctly reflect the variance in the within-subject design (Morey, 2008). **(Right)** Paired observation of two forces.

The two-way interaction of touch by force conditions was significant (F_(3,4486)_ = 5.48, *p* < 0.001), indicating that, independently from the effect of muscle, in the <∼1N force condition, MEPs were larger for Self-Touch (*M* = 0.79, SD = 0.31, *p* = 0.01), and We-Touch (*M* = 0.78, SD = 0.12, *p* = 0.02) compared to No-Touch (*M* = 0.66, SD = 0.22). In the ∼3N force condition, MEPs were larger for Self-Touch compared to both Surface Touch (*M* = 1.12, SD = 0.48, and *M* = 0.87, SD = 0.35, *p* < 0.001) and No-Touch (*M* = 1.12, SD = 0.48, and *M* = 0.84, SD = 0.25, *p* < 0.001), and in the same vein, for We-Touch compared to both Surface Touch (*M* = 1.12, SD = 0.32, and *M* = 0.87, SD = 0.35, *p* < 0.001) and No-Touch (*M* = 1.12, SD = 0.32, and *M* = 0.84, SD = 0.254, *p* < 0.001) (see Figure 4).

**FIGURE 5:**
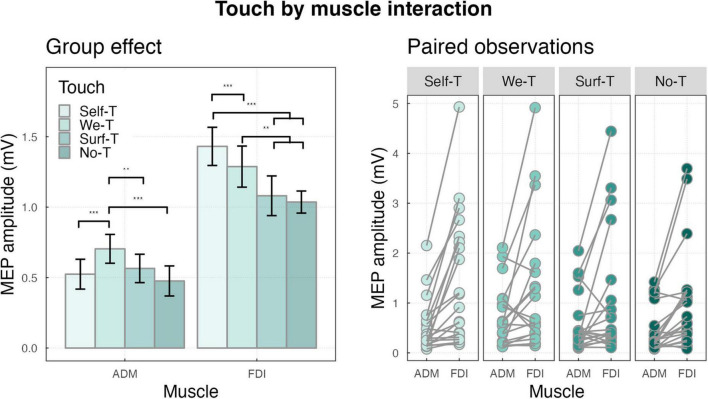
**(Left)** Bar plot showing the group effects of touch by muscle interaction ***P < 0.001 and **P < 0.01. Error bars represent the standard error of the means adjusted to correctly reflect the variance in the within-subject design (Morey, 2008). **(Right)** Paired observation of two muscles ADM and FDI in all touch conditions.

The interaction touch by muscle was significant (F_(3,4485)_ = 21.11, *p* < 0.001), for ADM muscle, MEPs were larger for the We-Touch condition compared to the Self-Touch condition (*M* = 0.70, SD = 0.46, and *M* = 0.52, SD = 0.47 *p* < 0.001), compared to the Surface-Touch condition (*M* = 0.70, SD = 0.46, and *M* = 0.56, SD = 0.45, *p* = 0.003), and compared to the No-Touch condition (*M* = 0.70, SD = 0.46, and *M* = 0.48, SD = 0.48, *p* < 0.001). For FDI muscle, MEPs were larger for the Self-Touch condition compared to the We-Touch condition (*M* = 1.43, SD = 0.28, *M* = 1.29, SD = 0.65, *p* < 0.001), compared to the Surface Touch condition (*M* = 1.43, SD = 0.28, *M* = 1.08, SD = 0.63, *p* < 0.001), and compared to the No-Touch condition (*M* = 1.43, SD = 0.28, *M* = 1.04, SD = 0.35, *p* < 0.001), MEPs were also larger for the WT condition compared to the Surface Touch condition (*M* = 1.29, SD = 0.65, *M* = 1.08, SD = 0.63, *p* = 0.002), and the No-Touch condition (*M* = 1.29, SD = 0.65, *M* = 1.04, SD = 0.35, *p* < 0.001) (see Figure 5).

The three-way interaction, touch by force by muscle, just approached significance (F _(3,4485)_ = 2.48, *p* = 0.06).

### Correlation

**FIGURE 6:**
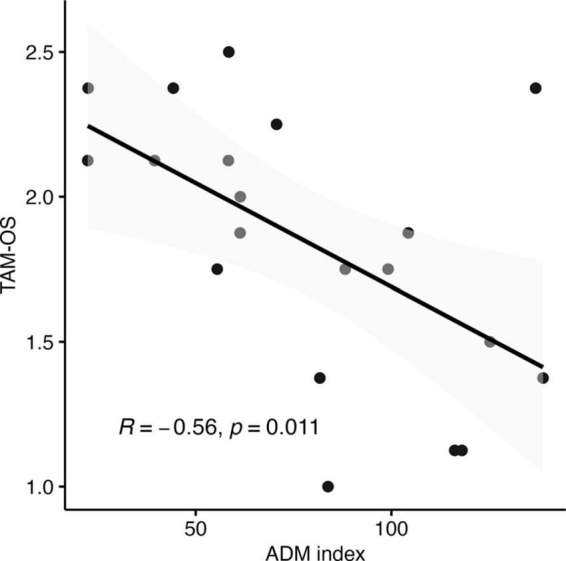
Correlation between the touch avoidance questionnaire in opposite sex TAM-OS score and the model predictions for the ∼3N force effect of ADM muscle.

Results of the Pearson correlation indicated that there was a significant negative correlation between the “Self-We Touch effect” -corresponding to the ratio between Self-Touch and We-Touch in the ∼3N force condition, for the ADM muscle activity - and the touch avoidance measure score, r (18) = −0.56, *p* = 0.01. The finding indicates that individuals with a low index (therefore, individuals in which We-Touch was higher compared to Self-Touch) were associated with a higher level of touch avoidance (i.e., individuals with a high score in the touch avoidance measure) (see Figure 6).

## Discussion

Our study aimed at investigating the neural cortico-spinal activity changes during action imagination when touch is involved. Specifically, we focused on examining the motor system activity during the mental simulation of force generation while individuals experienced various surfaces’ touch The experimental conditions examined included Self-Touch (touching each other with the two index fingers of the two hands), We-Touch (being touched by another person), Surface Touch (touching a surface with the index finger), and a condition without any tactile involvement i.e., No-Touch. By employing single-pulse transcranial magnetic stimulation (TMS) on the primary motor cortex, we specifically targeted the First Dorsal Interosseous (FDI) as the muscle responsible for the force production through the index finger pressure imagination, and the Abductor Digiti Minimi (ADM) serving as control muscle. Our study sought to provide valuable insights into the modulation of the motor system associated with different contexts in which social and physiological components are intermingled. We utilized imagination instead of actual action to avoid potential confounding factors, and to ensure a more controlled and focused investigation into the distinct contributions of the sensory component (encompassing biological and non-biological touch) and the motor component (involving the application of physical force). This well-established approach is based on the substantial similarities between action and action imagination on psychophysical properties, and patterns of neural activations (Decety et al., 2001; Papaxanthis et al., 2002; Ehrsson et al., 2003; Lotze and Halsband, 2006; McAvinue and Robertson, 2008; Munzert et al., 2009; Hétu et al., 2013; Zabicki et al., 2017).

The results confirmed our main hypothesis that the motor system excitability is enhanced when biological sources are touched, i.e., when individuals are either touching themselves and when touching someone else, as compared to when they are touching non-living sources, or not touching at all. We also replicated previous findings showing higher activation when individuals produce higher force as compared to lower force (Ali et al., 2023), and, as expected, the effect was stronger for the target muscle. The implications of these findings are considered in detail in the following discussion.

Overall, the amplitude of motor-evoked potentials (MEPs) of the relevant muscle increased when imagined actions were performed concurrently with tactile stimulation, replicating the facilitatory effect of touch on the motor system (Mizuguchi et al., 2013; Ali et al., 2023). During the action, there is a complex interplay between the somatosensory system and the motor system to jointly control the movement and process tactile information (see review Delhaye et al., 2016). On one hand, action allows to optimize of tactile precision through various mechanisms, such as the adjustment of movement parameters and predictive processes (Lederman and Klatzky, 1987; Chapman, 1994; Srinivasan and LaMotte, 1995; Louw et al., 2000; Gamzu and Ahissar, 2001; Giachritsis et al., 2009; Kaim and Drewing, 2011; Drewing, 2012; Lezkan and Drewing, 2014; Metzger et al., 2018). On the other hand, somatosensory information is integrated with other processes to control action. However, how exactly this interplay is implemented in the brain needs to be addressed. Here we showed that the processing of tactile information directly impacts M1 excitability during motor imagery.

We found increased activation of the motor system when participants imagined producing force (∼3N) as compared to no-force production (<∼1). Importantly, the scaling effect of force was larger for the target muscle, i.e., FDI, compared to the control muscle, i.e., ADM. That is the increased motor system activation when imaging to produce ∼3N compared to <∼1N was significantly more pronounced for FDI, the muscle that would be involved in the execution of the action (Bufalari et al., 2010; Pizzolato et al., 2012; Helm et al., 2015; Ali et al., 2023; Figure 3). It is well established that M1 neurons encode grasp force (Cheney and Fetz, 1980; Wannier et al., 1991; Maier et al., 1993; Sergio and Kalaska, 1997, 1998; Shalit et al., 2012) and carry relevant information pertaining to grasp force (Intveld et al., 2018). In line with Ali et al. (2023), the present finding suggests that forces applied during imagined actions are scaled similarly to physical actions and that the scaling is selective for the body part involved (Bufalari et al., 2010; Pizzolato et al., 2012; Helm et al., 2015; Grosprêtre et al., 2016), even when a modest magnitude of force is employed [∼3N in the present investigation versus ∼15N in Ali et al. (2023)]. This scaling effect is consistent with earlier research that explored various imagery tasks, such as grasping objects of different sizes and shapes, performing movements in different directions, and varying the extent of movement (Bufalari et al., 2010; Cesari et al., 2011; Pizzolato et al., 2012).

The ability to discriminate physical properties through touch is accompanied by the integration of affective and socially relevant information. While studies over the past decades have provided important information about the reciprocal interplay between touch and action in general (Camerota and Celletti, 2014; Moscatelli et al., 2019), how social touch impacts movement control remains to be determined. Another important finding of the present study was the identification of how Self-Touch and We-Touch modulate the MEPs’ amplitude during force imagination. In the condition (<∼1), both Self-Touch and We-Touch showed higher activation compared to No-Touch, while there was no significant difference among the three touching conditions. In other words, the M1 excitabilities in the Self-Touch, the We-Touch, and the Surface Touch conditions were similar. On the other hand, when subjects were imaged to produce force (∼3N force condition), the excitability of M1 in Self-Touch and We-Touch conditions was similar, both showing higher excitability than Surface Touch and No-Touch. Therefore, it appears that while the main facilitatory effect of touch was present even in the absence of action, the presence of action appeared critical to differentiate between touches when in contact with “living” and “not-living” materials. This finding strongly corroborates previous studies that have associated improved tactile perception with action, possibly attributed to the influence of predictive processes (Smith et al., 2009; Willemet et al., 2021).

Critically, the differences among the types of touch were manifest when comparing the patterns of activation of the two muscles. For the target muscle, i.e., FDI, both Self-Touch and We-Touch showed larger amplitude compared to Surface Touch and No-Touch. However, in line with previous evidence indicating that self-touch and social touch are associated with different patterns of neural activation (Boehme et al., 2019), we found that MEPs’ amplitude in the Self-Touch condition was significantly larger than the amplitude in the We-Touch condition. That is, the motor system activation was the highest when the right index finger of the subject was in contact with her own left index finger. This result sustains the hypothesis of the presence of sensory attenuation (SA) explaining why stimuli that are self-generated are associated with a reduction in the perceived intensity of the stimulus, explaining for instance why one cannot tickle oneself (Hughes et al., 2013). As a result, the discrimination between tactile signals produced by the action of the same person and signals that arise from non-self-causes is hypothesized to be based on this mechanism of SA (Blakemore et al., 1998; Master and Tremblay, 2010). When a motor command is generated, an internal copy of this command, referred to as an “efference copy,” is utilized to predict, and subsequently attenuate, the sensory outcome of the action (e.g., Kilteni et al., 2020). Consequently, self-generated tactile sensations are perceived as weaker compared to externally imposed stimuli (Bays and Wolpert, 2012). Therefore, it is tempting to explain the highest activation found in the Self-Touch condition for the target muscle as a neural signature of the consequences of sensory attenuation. Specifically, since the brain might have predicted the specific action (right index finger touching the left index finger) through an efferent copy, the sensory signal associated with the right hand could have been attenuated. Consequently, the amount of force “exerted” by the right index finger to match the required force (∼3N) might have been exaggerated. However, further research should be carried out to establish the validity of this hypothesis.

In stark contrast with FDI, the motor system excitability for the control muscle, i.e., ADM, was highest in the We-Touch condition, that is when the subject’s right index finger was in contact with the index finger of another individual. The We-Touch condition showed a significantly larger amplitude compared to all the other conditions (Self-Touch, Surface Touch, and No-Touch), while among those, no significant difference was detected. Considering that ADM was not directly involved in the action (Ali et al., 2023), this finding confirms that affective touch may have a more pervasive influence compared to other types of touch, aligning with the idea that the brain is hardwired around social dimensions (Dunbar, 2010), and that, social cues and contexts are processed as exceptionally influential information (Souza et al., 2012; Grichtchouk et al., 2022). Affective touch is mediated by a specialized sub-modality of touch, the c-touch system (McGlone et al., 2014), and fMRI evidence suggests that social touch is associated with widespread neural activity (Boehme et al., 2019). In line with that, the We-Touch condition may have engaged a specific and broader neural pathway encompassing ADM control.

Alternatively, some insight into the underlying mechanism of this effect may come from the correlation analysis that we carried out between the MEPs amplitude and the outcomes of the Touch Avoidance Measure (Casetta et al., 2020). We computed an index for each of the two muscles separately (ratio between Self-Touch and We-Touch in the ∼3N force condition) to better identify the difference between the Self-Touch and the We-Touch condition, which we refer to as the “Self-We touch effect.” Remarkably, we found a significant negative association between the ADM index and the scores on the Touch Avoidance Measure specifically concerning participants’ comfort levels when touching someone of the opposite sex. Individuals who exhibited higher ADM activation in the other touch condition (lower Self-Touch and We-Touch ratio) were associated with a higher score in the Touch Avoidance Measure. In other words, participants who showed a tendency toward touch avoidance, as measured by the Touch Avoidance Measure, also showed higher MEPs in the We-Touch condition compared to the Self-Touch condition. The touch avoidance construct serves as an indicator of an individual’s inclination toward initiating and receiving physical touch (Andersen and Leibowitz, 1978). Touch avoidance has been shown to diminish the perceived pleasantness of various forms of tactile stimulation (Hielscher and Mahar, 2017). The primary function of ADM is to move the fifth finger away from the fourth finger, and therefore, it is involved in each movement that requires the spreading or abduction of the little finger from the ring finger. Therefore, another possible explanation for the “Self-We Touch effect” for ADM is that it reflects the intention to move the hand far away to avoid the somewhat unpleasant sensation associated with touching a stranger. Future work might focus on the Self-We Touch differences, systematically varying parameter such as force magnitude and/or movement required and muscle involvement, to determine whether there is a causal link between the personality trait related to touch avoidance and specific muscle activation.

In conclusion, the purpose of the current investigation was to clarify the role of different types of touch during action imagination. In line with our previous study, touch was associated with higher M1 excitability, and as expected, the effect scaled with the force “exerted” and was selective for the target muscle. Crucially, while in the absence of force production imagination, there was just a general effect of touch, when subjects imagined producing force, the effects of different types of touch become manifest. Touch conditions were arranged in a somewhat hierarchical order, with Self-Touch having the greatest impact, followed by We-Touch, Surface Touch, and finally, No-Touch, which exhibited the lowest amplitude of MEPs. Finally, the control muscle (ADM), showed increased activation during the We-Touch condition, which we attributed to the strong effect of the social context or as a possible inclination to avoid touching an unfamiliar person.

The present study contributes to our understanding of the integration between sensory and motor processes, and the reciprocal interplay between touch and action. Overall, the study strengthens the idea that action facilitates sensory discrimination, allowing better differentiation among different types of touch. We provide evidence in favor of the hypothesis that self-other discrimination is based on a mechanism of sensory attenuation. In addition, the study has been one of the first attempts to thoroughly examine how social touch impacts motor control at the level of motor system activation.

## Data availability statement

The raw data supporting the conclusions of this article will be made available by the authors, without undue reservation.

## Ethics statement

The studies involving humans were approved by the Ethical committee (code number n.28.R1/2021) of the University of Verona. The studies were conducted in accordance with the local legislation and institutional requirements. The participants provided their written informed consent to participate in this study.

## Author contributions

YA: Data curation, Formal analysis, Methodology, Software, Writing – original draft, Writing – review & editing, Investigation. VM: Data curation, Formal analysis, Investigation, Methodology, Software, Writing – original draft, Writing – review & editing. PC: Data curation, Formal analysis, Methodology, Software, Writing – original draft, Writing – review & editing, Conceptualization, Project administration, Supervision, Validation, Visualization.
